# Decreased Pattern Recognition Receptor Signaling, Interferon-Signature, and Bactericidal/Permeability-Increasing Protein Gene Expression in Cord Blood of Term Low Birth Weight Human Newborns

**DOI:** 10.1371/journal.pone.0062845

**Published:** 2013-04-23

**Authors:** Vikas Vikram Singh, Sudhir Kumar Chauhan, Richa Rai, Ashok Kumar, Shiva M. Singh, Geeta Rai

**Affiliations:** 1 Department of Molecular and Human Genetics, Faculty of Science, Banaras Hindu University, Varanasi, India; 2 Department of Pediatrics, Institute of Medical Sciences, Banaras Hindu University, Varanasi, India; 3 Department of Biology, The University of Western Ontario, London, Ontario, Canada; Centre d'Immunologie de Marseille-Luminy, CNRS-Inserm, France

## Abstract

**Background:**

Morbidity and mortality rates of low birth weight (LBW) newborns at term are higher than rates in normal birth weight (NBW) newborns. LBW newborns are at greater risk to acquire recurrent bacterial and viral infections during their first few weeks of life possibly as an outcome of compromised innate immune functions. As adaptive immunity is in a naive state, increased risk of infection of LBW as compared to NBW newborns may reflect impairments in innate immunity.

**Methodology:**

To characterize the increased susceptibility to infections in LBW newborns we used microarray technology to identify differences in gene expression in LBW newborns (n = 8) compared to NBW newborns (n = 4) using cord blood. The results obtained from the microarray study were validated on a larger number of samples using real time RT-PCR (LBW = 22, NBW = 18) and western blotting (LBW = 12, NBW = 12). The Interferome database was used to identify interferon (IFN) signature genes and ingenuity pathway analysis identified canonical pathways and biological functions associated with the differentially expressed genes in LBW newborns. ELISAs for IFNs and bactericidal/permeability-increasing protein were performed in both LBW and NBW newborns and in adults (LBW = 18, NBW = 18, Adults  = 8).

**Principal Findings:**

Upon microarray analysis, we identified 1,391 differentially expressed genes, of which, 1,065 genes were down-regulated and 326 genes were up-regulated in the LBW compared to NBW newborns. Of note, 70 IFN-signature genes were found to be significantly down-regulated in LBW compared to NBW newborns. Ingenuity pathway analysis revealed pattern recognition receptors signaling including Toll-Like Receptors (TLRs) -1, -5, and -8 genes and IFN signaling as the most significantly impacted pathways. Respiratory infectious diseases were the most significantly affected bio-functions in LBW newborns.

**Conclusion and Significance:**

Diminished PRRs, IFN-signature, and BPI gene expression raises the possibility that impairments in these pathways contribute to the susceptibility of LBW term infants to infection.

## Introduction

Low birth weight (LBW) (birth weight <2500 g) newborns suffer from higher infection, morbidity and mortality rates than normal birth weight (NBW) (birth weight ≥2500 g) newborns [1]–[3]. Over 20 million newborns worldwide, representing 15.5% of all births, are LBW, of which, 95.6% are in developing countries [4]. LBW newborns may be either term LBW (intrauterine growth restriction; gestation age ≥37 weeks) or pre-term (gestation age <37 weeks) newborns. The percentages of term LBW newborns are very high with estimates of nearly 75% in Asia, 20% in Africa and approximately 5% in the Latin America [5]. India has highest number of LBW infants born each year, representing 40 percent of the global burden, of which, three-fourths are born at term [6]. LBW neonates have high morbidity from diarrhea and their risk of death is estimated to be four to ten times higher than NBW newborns [2], [7]. LBW newborns are also known to contract life-threatening diseases like pneumonia or acute lower respiratory infections at a rate almost twice than that of NBW newborns [8]. Differences in the immune responses between newborns and adults, and between preterm and term newborns, have been reported [9]–[12]. LBW, irrespective of the gestational age, may also influence immune mechanisms. However, the variations in the immune status between term LBW and NBW newborns is only partially understood as there are only a few studies available in this area which focus primarily on comparing immunoglobulin classes, complement levels, T- and B-cell counts. It has been shown that LBW newborns have lower levels of IgG [13], [14], [15], impaired early IgA and IgM synthesis [15], and lower T- and B- lymphocyte percentage [13], [16], [17] than the NBW newborns, whereas little is known about their potential differences in innate immunity. A larger picture comprising exact components and mechanisms involved in defective innate immune signaling in term LBW newborns remains yet to be drawn. We therefore undertook this study targeting the global gene expression to obtain a fuller view of distinct immune system of LBW newborns. We performed gene expression microarray analysis of cord blood cells and identified interferon stimulated genes (ISGs) significantly down-regulated in LBW newborns. *Pattern recognition receptor (PRR) signaling* and *IFN signaling* were the most significantly down-regulated in LBW newborns. Other significantly affected bio-functions and disease categories in the LBW newborns were *infectious disease*, *respiratory diseases, antimicrobial response, inflammatory response*, *antigen presentation*, *hematological system development and functions*, and *immune cell trafficking.* In addition, many immune response related genes like granzymes and bactericidal/permeability-increasing (BPI) protein were also seen to be down-regulated in the LBW newborns. Further, the IFNs and BPI protein were also confirmed to be less produced in response to toll-like receptor (TLR)-8 signaling in LBW newborns. The information obtained from this study may provide us with a better understanding of the underlying mechanisms responsible for the underdeveloped immune response in the term LBW newborns and their increased susceptibility to infections. Further in-depth investigations on the novel target molecules identified through this study may pave the way for the development of more precise and targeted therapies that can protect the LBW newborns from life threatening infections.

## Materials and Methods

### Ethics statement

Ethical consent for use of human material was obtained from the institutional research ethics committee for this study. Informed and written consent were taken from the parents of each newborn enrolled in the study.

### Subjects, sample collection, and clinical assessment

Cord blood from 79 full-term newborns (39 NBW and 40 LBW) and peripheral blood from 8 adults (Table S1) was collected in sterile tubes containing non-pyrogenic anti-coagulant sodium heparin from the Department of Pediatrics, Sir Sunder Lal Hospital, Banaras Hindu University, Varanasi, India. Ethical consent for use of human material was obtained from the institutional research ethics committee for this study. Informed and written consent were taken from the parents of each newborn enrolled in the study. Birth weights were measured with great care and precision, and all newborns were weighed immediately after birth. Births in which antibiotics were administered during intrapartum period were excluded from the study. Detailed information pertaining to mother and her baby was collected on a predesigned questionnaire. The information regarding birth weight, gender, gestational age and type of delivery of a baby is provided in Table S1.

### RNA extraction from cord blood samples

Heparinized cord blood samples taken from LBW and NBW newborns were diluted with 4 volumes of RBC lysis buffer (155 mM NH_4_Cl, 10 mM KHCO_3_, 0.1 mM EDTA, pH 7.4) and incubated for 15 minute at 37°C in 5% CO_2_ and centrifuged at 400 X g, for 5 min at room temperature to pellet leukocytes. After discarding the supernatant, leukocytes were then washed twice with PBS and lysed using TRI-reagent (Sigma-Aldrich) for RNA extraction as per manufacture's protocol. Further, total RNA was subjected to DNase treatment for removal of genomic DNA contamination. All RNA samples were quantified using a ND-1000 spectrophotometer (NanoDrop Technologies) and their quality were assessed using 2100 Bioanalyzer (Agilent Technologies).

### RNA labeling and Affymetrix gene chip expression probe array hybridization

Genome wide mRNA expression was determined using cord blood leukocytes from eight LBW and four NBW newborns (Table S1). Biotinylated cRNA was prepared from 100 ng of total RNA as per the Affymetrix gene chip expression analysis technical manual. Briefly, 100 ng of total RNA was used to synthesize first-strand cDNA primed with T7 oligo (dT)_24_ primer followed by second-strand cDNA synthesis using the one cycle target labeling kit (Affymetrix) to produce double stranded cDNA. After second-strand synthesis, the cDNA was purified with the GeneChip sample cleanup module (Affymetrix). Further, in-vitro transcription of biotin-labeled cRNA from double-stranded cDNA was carried out using the GeneChip IVT labeling Kit (Affymetrix). The biotin-labeled cRNA were further, purified, fragmented, and hybridized (10 µg) on GeneChip Human Genome U133 Plus 2.0 Array (Affymetrix) for 16 hrs at 45°C. The hybridized probe arrays were washed and stained with Streptavidin-Phycoerythrin (Molecular Probes), followed by biotinylated anti-streptavidin for linear amplification of signals (Vector Laboratories). The Arrays were scanned by an Affymetrix GeneChip® Scanner 3000 at 570 nm using the GeneChip Operating Software (GCOS).

### Microarray Data Analysis

The intensity values of different probe sets (genes) generated by Affymetrix GCOS were imported into GeneSpring GXv11.0 software (Agilent Technologies) for raw data summarization and normalization. The data files (CEL files) containing the probe level intensities were pre-processed by GeneChip Robust Multichip Average (GC-RMA), according to the gene information from the array. The GC-RMA analyses converted the probe-level expression data into gene-level expression data and quintile normalization was performed. Baseline transformation of LBW samples (E1 to E8) was done with respect to control NBW samples (C1 to C4). A difference of at-least two fold in the gene expression between NBW and LBW sample were considered for further analysis. P-values were derived based on Student's t-test and Benjamin-Hocheberg false discovery rate tests for each of the differentially regulated genes across the biological replicates. The complete sets of raw and normalized data from this study have been deposited in the Gene Expression Omnibus repository (GEO series accession number: GSE29807).

### Interferome Database

Interferome is an open access database providing information concerning type I, II and III IFN regulated genes [18]. Differentially expressed genes identified by Gene Spring were imported into the ‘Interferome’ database (http://www.interferome.org/) to identify the ISGs in LBW newborns.

### Pathways, Bio-functions, and Networks analysis

Accession numbers for all differentially expressed genes that displayed a minimum of 2-fold change were imported into Ingenuity Pathway Analysis (IPA) (http://www.ingenuity.com/) for further data analysis. IPA constructed hypothetical gene/protein interactions between our sets of differentially expressed genes refered as the ‘focus gene’ and all other genes stored in the knowledge base. These focus genes in LBW newborns were selected for generating canonical pathways, bio-functions, and networks. Canonical pathway analysis identified the pathways that were most significant in the data set. The significance of the association between the data set and the canonical pathway was measured using a ratio of the number of genes from the data set that map to the pathway to the total number of genes that map to the canonical pathway and Right-tailed Fisher's exact test. The networks with the highest scores and focus molecules were identified by IPA network analysis and displayed graphically as a collection of nodes (genes/gene products) and edges (the biological relationships between the nodes). The intensity of the node colors indicates the degree of up-regulation (red) or down-regulation (green). Nodes are displayed using various shapes that represent the functional class of the gene product. Edges are displayed with various labels that describe the nature of the relationship between the nodes. The score is derived from a p-value and indicates the likelihood of the focus genes in a network being found together because of random chance. A score of 2 or higher indicates at least a 99% confidence level of not being generated by random chance alone. The biological functions are then calculated and assigned to each network.

### Real time reverse transcription-polymerase chain reaction (RT-PCR)

The RNA from cord blood of 22 LBW and 18 NBW (Table S1) newborns was used for quantitative real time RT-PCR assay. Reverse transcription of 1 µg of total RNA per reaction was performed for cDNA synthesis using High-Capacity cDNA Reverse Transcription Kits (Applied Biosystem) as per manufacturer's protocol. Gene-specific primers for real time PCR were designed using Autoprime software (http://www.autoprime.de/AutoPrimeWeb) (Table 1). The PCR was conducted using Power SYBR Green PCR Master Mix (Applied Biosystems) and Quantifast SYBR Green PCR Kit (Qiagen). The real time PCR amplification was performed on ABI 7500 real-time PCR system (Applied Biosystems)/iQTM5 real-time PCR detection system (Bio-Rad). Each sample was run in duplicate. Real time PCR data were analyzed using the 2^−ΔΔCt^ method as described by Livak and Schmittgen [19]. The β-actin, a house keeping gene, was selected as an internal control. There was no significant difference in the Ct values of β-actin between LBW and NBW newborns. The results are expressed as fold change  = 2^−ΔΔCt^, where ΔΔCt  =  {Ct_target_ –Ct_β-actin_}_LBW_ – {Ct_target_ – Ct_β-actin_}_NBW_. Statistical significance was determined using Student's t-test from ΔCt values from LBW and NBW groups (p<0.05).

**Table 1:** Gene specific primers for real-time RT-PCR.

Gene	Forward primer	Reverse primer	Annealing emperature	Product size (base pair)
TLR-5	5′ACTCCTGATGCTACTGACAAC 3′	5′GTATAGCATCCCTGGTTTGG 3′	57°C	59
TLR-8	5′TTATGTGTTCCAGGAACTCAGAGAA 3′	5′TAATACCCAAGTTGATAGTCGATAAGTTTG 3′	58°C	82
IRF-7	5′CCTGGTGAAGCTGGAACC 3′	5′TGCTATCCAGGGAAGACACAC 3′	58°C	80
β-actin	5′CTTCCTGGGCATGGAGTC 3′	5′TACAGGTCTTTGCGGATGTC 3′	58°C	87

### Western blot analysis

Cord blood cells were collected from 12 NBW and 12 LBW newborns (Table S1). Whole blood leukocytes were obtained after lysis the RBCs using RBC lysis buffer. Further, leukocytes were lysed in RIPA buffer (Cell Signalling Technology). The lysates were centrifuged at 12,000 rpm for 15 min at 4°C and the soluble supernatants were stored at −80°C. The protein was quantified using Bradford method [20]. For Western blotting, 80 μg of cell protein was boiled for 5 min in 5X SDS sample buffer containing 100 mM of the reducing agent Di-thiothreitol. Electrophoresis was then carried out using 10% polyacrylamide gels. Proteins were then transferred to PVDF membrane (Thermo Scientific) at 30V/90 mA for overnight. Following transfer, blots were incubated with primary antibodies at the following dilutions: mouse anti-human TLR-5 (1:100), mouse anti-human TLR-8 (1:100) and rabbit anti-human GAPDH (1:1000) (Imgenex). Overnight incubation with primary antibody at 4°C was followed by 2 hrs incubation with rabbit anti-mouse (1:1000) and goat anti-rabbit (1:2000), horseradish peroxidase (HRP) secondary antibody conjugates (Santa Cruz Biotechnology). Proteins were detected using an enhanced chemiluminescence HRP substrate reagent (Millipore).

### Induction of BPI and IFN-α with R-848

Cord blood from 18 NBW, 18 LBW newborns, and peripheral blood from 8 adults was collected in sterile tubes containing non-pyrogenic sodium heparin (Table S1). The blood was diluted with equal volume of RPMI 1640 media and stimulated with R-848 (TLR-8 ligand;10 µg/ml, Enzo Life Sciences) in culture vials for 18 hrs at 37°C in 5% CO2 incubator. Following incubation the culture supernatant was harvested after centrifugation and stored at −80°C to be later assayed for BPI protein and IFN-α by ELISA.

### Quantitative measurement of Human IFN-α and Human BPI by ELISA

#### IFN-α ELISA

The concentration of IFN-α released by cord blood cells of the newborns and peripheral blood cells of the adults in response to R-848 was measured using sandwich Human IFN-α multi-subtype ELISA Kit (PBL Interferon Source) according to manufacturer's protocol. The assay was performed in duplicate for each sample. Briefly, standards and sample were added to the wells pre-coated with antibody IFN-α. The plate was incubated at room temperature for 1 hr followed by removal of samples and washing one times with wash buffer. Diluted IFN-α antibody was added to wells and incubated for 1 hr followed by three times washing with wash buffer. HRP conjugate was diluted and pipetted to each well. The plate was incubated at room temperature for 1 h followed by 4 times washing with wash buffer. TMB Substrate was added into each well and plate was allowed to develop for 15 minutes followed by addition of stop solution. The absorbance of samples was read at 450 nm in an ELISA plate reader (BioRad). A 4-parameter best fitting standard curve was generated to determine the concentration of IFN-α protein.

#### BPI ELISA

The concentration of BPI released in response to R-848 was measured using sandwich Human BPI ELISA Kit (Hycult Biotech) according to manufacturer's protocol. The assay was performed in duplicate for each sample. Briefly, wells were washed 4 times with wash buffer followed by addition of standards and sample to the pre-coated wells with antibody BPI. The plate was incubated at room temperature for 2 hrs followed by removal of samples and washing one time with wash buffer. Biotinylated tracer was added to wells and incubated for 1 hr followed by 4-times washing. Streptavidin-peroxidase enzyme was diluted and pipetted to each well. The plate was incubated at room temperature for 1 hr followed by 4 times washing with wash buffer. TMB Substrate was added into each well and plate was allowed to develop for 30 minutes followed by addition of stop solution. The OD of samples was read at 450 nm in an ELISA plate reader (BioRad). A 4-parameter best fitting standard curve was generated to determine the concentration of BPI protein.

### Statistical Analysis

The mRNA and protein expression of TLR-5, TLR-8 and IRF-7 were compared using Student's t test. Culture supernatant concentration of IFN-α and BPI protein in adults, NBW and LBW newborns were compared using the Kruskal-Wallis test. The statistical analysis was performed on raw or logarithmically transformed data whichever was appropriate. All statistical tests were performed by Prism software for Windows or Microsoft Office Excel 2007. The level of significance was determined by p-value <0.05 in all comparisons.

## Results

### Identification of differentially expressed genes in LBW newborns

**Figure 1:**
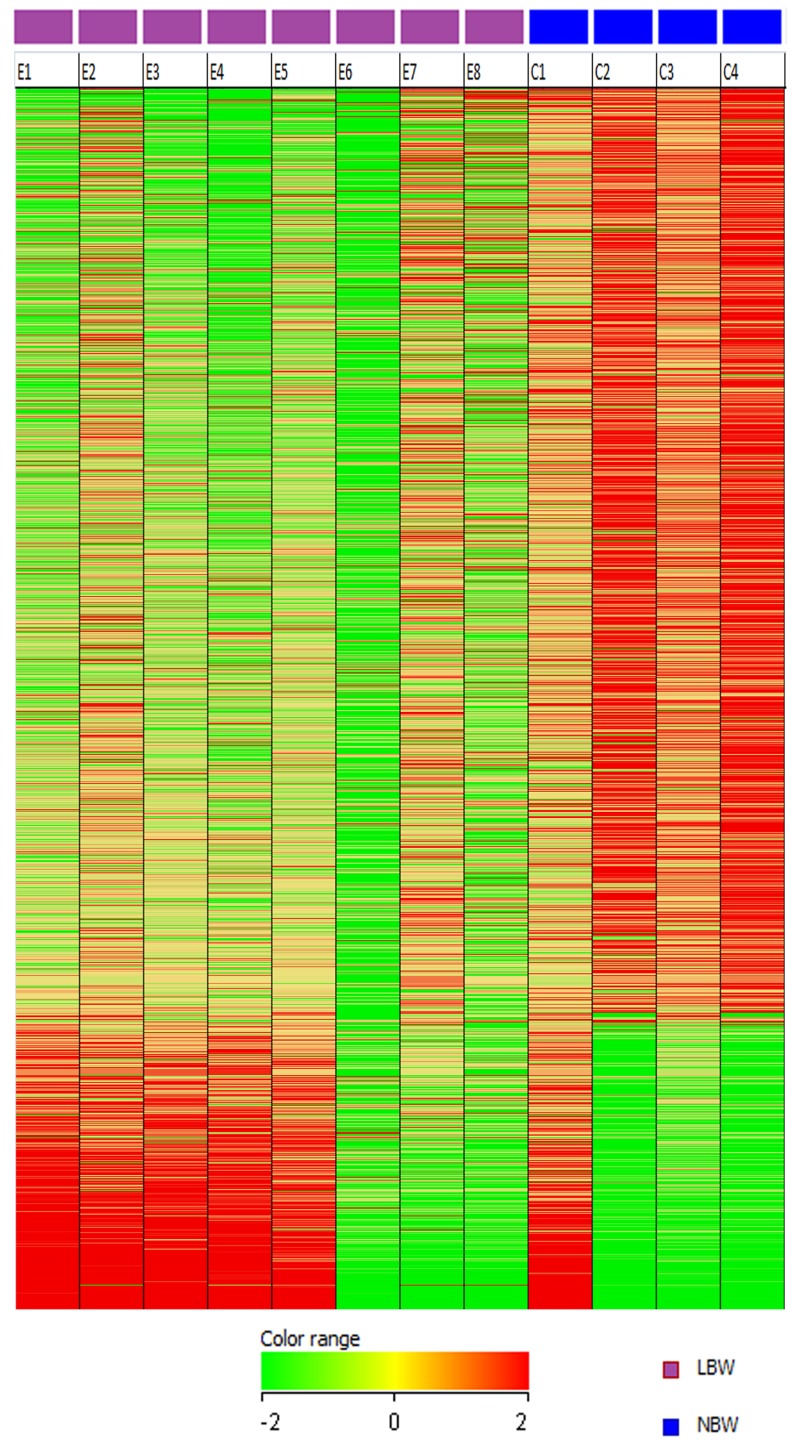
Expression profile of 1391differentially expressed genes in LBW and NBW newborns. Gene expression profiles are displayed as a heat-map where the columns correspond to samples (LBW; E1–E8 and NBW; C1–C4) and the rows correspond to genes. Red represents higher relative expression and green represents lower relative expression.

We conducted gene expression analysis using GeneChip Human Genome U133 Plus 2.0 Array for whole genome expression profile of the LBW newborns. A total of 1391 differentially expressed genes having two-fold or greater differences in expression were identified, of which, 1065 genes were down-regulated and 326 genes were up-regulated in LBW newborns (Table S2). Down-regulation of genes appeared to be a more dominant event compared to up-regulation in LBW newborns (Figure 1) although the expression pattern of C1 (NBW) appears similar to that of E2 (LBW). The differentially expressed genes were further subjected to Interferome data base to identify the IFN-signature genes also referred to as ISGs. All the 1391 differentially expressed genes and ISGs distinguishing the LBW from NBW newborns were further subjected to IPA analysis to identify the pathways and biological functions associated with them.

### Down-regulated data set analysis

#### Gene expression analysis reveals significantly down-regulated ISGs in LBW newborns

**Figure 2:**
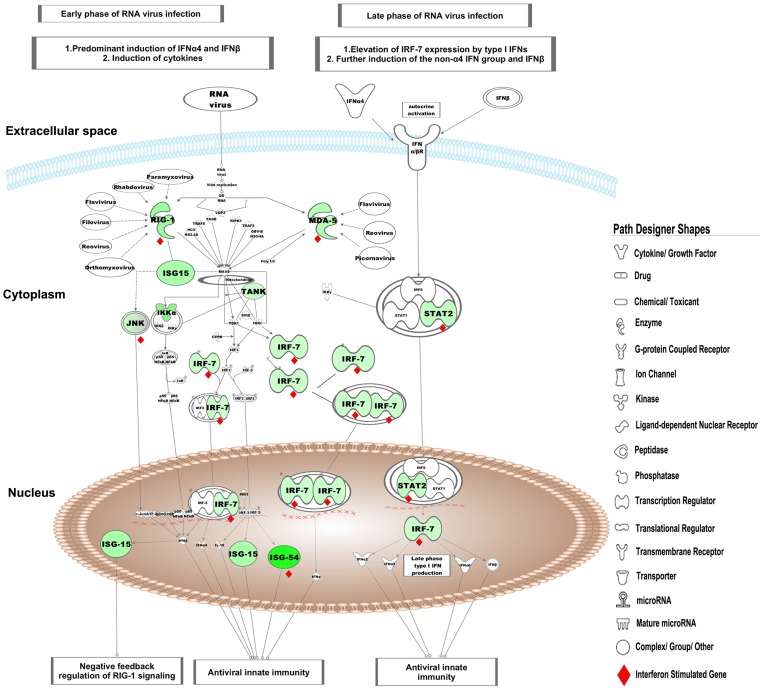
Activation of IRF by cytosolic pattern recognition receptor pathway. The green nodes in this canonical pathway indicate the down-regulated genes in LBW newborns. The uncolored nodes represent the genes integrated by IPA from its knowledge base. The interferon stimulated genes in this pathway are marked as red diamond (♦).

**Figure 3:**
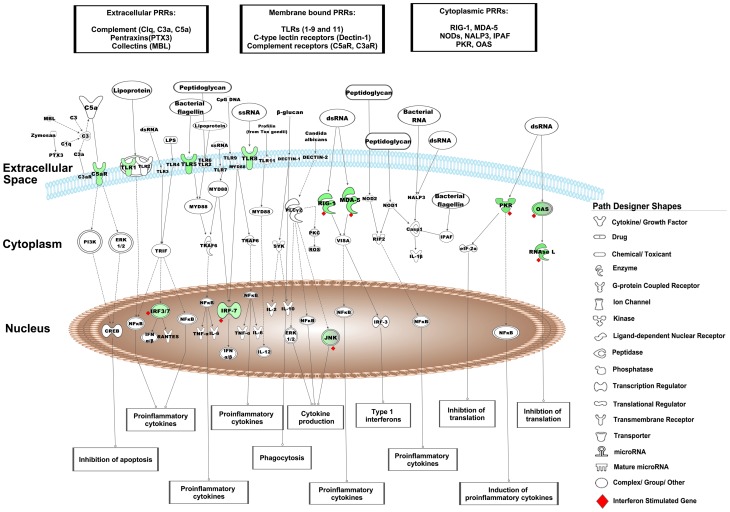
Role of pattern recognition receptors in recognition of bacteria and viruses pathway. The canonical pathway indicates the down-regulated genes in LBW newborns as green nodes. The uncolored nodes are the genes inferred by IPA from its knowledge base. The interferon stimulated genes in this pathway are marked as red diamond (♦).

**Figure 4:**
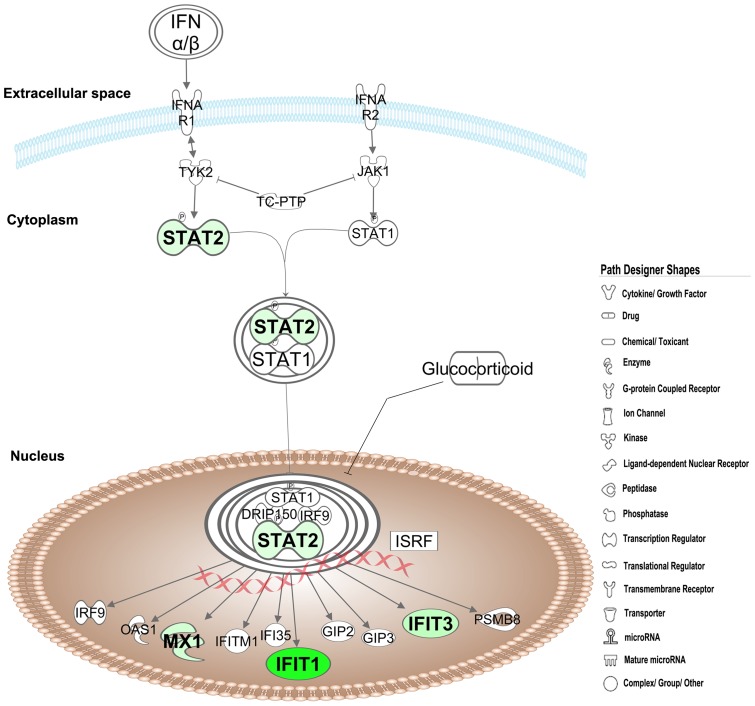
Interferon signaling pathway. The down-regulated ISGs in LBW newborns are shown in green color. The uncolored nodes are the genes inferred by IPA from its knowledge base.

**Figure 5:**
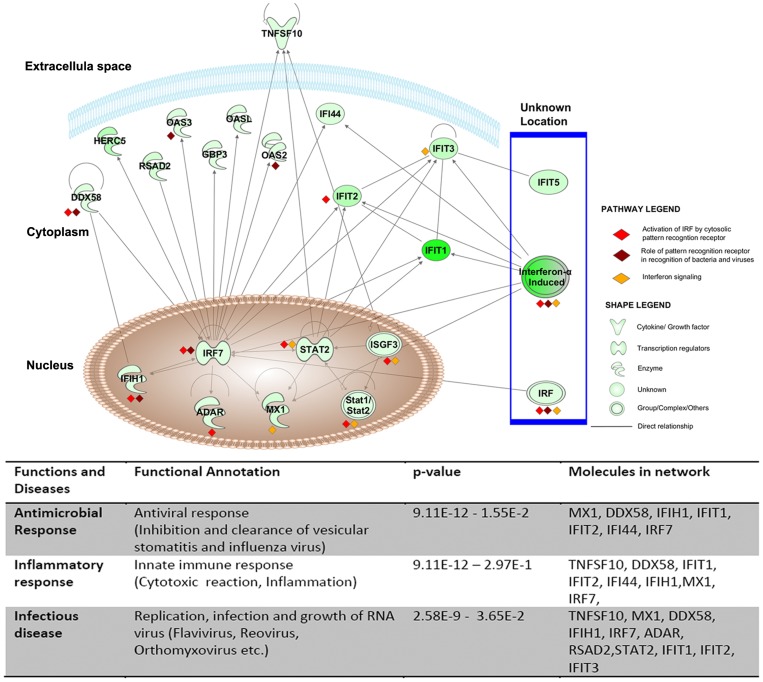
Top network of down-regulated ISGs in LBW newborns. The shapes legend classifies the proteins found as cytokines/growth factors, transcriptional regulators, enzymes and other groups. The pathway legend identifies genes that were present in the listed pathways that were down-regulated in the LBW newborns. The connecting lines indicate direct interactions among the products of these genes.

Analysis of the differentially expressed genes by the Interferome database identified 74 ISGs of which, 70 were significantly down-regulated and 4 were up-regulated (Table 2). Of the 70 down-regulated ISGs, 57 were annotated as type I IFNs by the Interferome data base (data not shown). We found that interferon-induced protein with tetratricopeptide repeats (IFIT)-1,-2,-3 and-5 were amongst the most down-regulated genes in LBW newborns. IPA analysis of these 70 down-regulated ISGs identified the most significantly associated pathways as i)*‘activation of interferon regulatory factors (IRFs) by cytosolic pattern recognition receptors’* (Figure 2), ii)*‘role of pattern recognition receptors in recognition of bacteria and viruses’* (Figure 3), and iii)*‘IFN signaling’* (Figure 4). Furthermore, the top most ISGs network generated by IPA was involved in biological functions like *antimicrobial response, inflammatory response* and *infectious disease* in the LBW newborns (Figure 5). It is noteworthy to mention that interferon regulatory factor-7 (IRF-7), signal transducer and activator of transcription-2 (STAT-2), and ISG-15 ubiquitin-like modifier-15 (ISG-15) of this network were the most significantly affected transcription factors in the LBW newborns.

**Table 2.Interferon:** stimulated genes in LBW newborns.

Affymetrix ID	Gene Symbol	p- Value	FC	RG	Affymetrix ID	Gene Symbol	p-Value	FC	RG
216508_x_at	HMGB1	0.0477	3.16	Up	221044_s_at	TRIM34	0.0080	2.53	Down
207113_s_at	TNF	0.0246	2.32	Up	204211_x_at	EIF2AK2	0.0070	2.47	Down
241930_x_at	PTPN11	0.0185	2.13	Up	212372_at	MYH10	0.0076	2.46	Down
201267_s_at	PSMC3	0.0479	2.05	Up	201589_at	SMC1A	0.0035	2.39	Down
203153_at	IFIT1	0.0039	27.03	Down	205170_at	STAT2	0.0051	2.39	Down
217502_at	IFIT2	0.0066	8.91	Down	201872_s_at	ABCE1	0.0404	2.38	Down
219863_at	HERC5	0.0054	8.73	Down	216994_s_at	RUNX2	0.0192	2.38	Down
204747_at	IFIT3	0.0188	7.45	Down	204641_at	NEK2	0.0184	2.36	Down
209585_s_at	MINPP1	0.0003	6.43	Down	204444_at	KIF11	0.0308	2.32	Down
204439_at	IFI44L	0.0155	5.95	Down	204026_s_at	ZWINT	0.0161	2.31	Down
203595_s_at	IFIT5	0.0022	5.83	Down	204769_s_at	TAP2	0.0425	2.28	Down
226603_at	SAMD9L	0.0005	5.51	Down	219691_at	SAMD9	0.0338	2.27	Down
213797_at	RSAD2	0.0468	5.24	Down	229723_at	TAGAP	0.0177	2.23	Down
202086_at	MX1	0.0041	5.12	Down	206925_at	ST8SIA4	0.0229	2.21	Down
207500_at	CASP5	0.0087	4.47	Down	223342_at	RRM2B	0.0340	2.19	Down
214059_at	IFI44	0.0271	4.47	Down	203213_at	CDC2	0.0107	2.16	Down
220646_s_at	KLRF1	0.0240	4.44	Down	223591_at	RNF135	0.0357	2.15	Down
227609_at	EPSTI1	0.0009	4.41	Down	203218_at	MAPK9	0.0219	2.14	Down
218400_at	OAS3	0.0097	4.17	Down	206715_at	TFEC	0.0479	2.14	Down
218943_s_at	DDX58	0.0104	3.79	Down	204804_at	TRIM21	0.0277	2.13	Down
225415_at	DTX3L	0.0024	3.39	Down	209707_at	PIGK	0.0135	2.11	Down
206785_s_at	KLRC1	0.0315	3.39	Down	200934_at	DEK	0.0147	2.1	Down
206637_at	P2RY14	0.0006	3.37	Down	208436_s_at	IRF7	0.0287	2.1	Down
205660_at	OASL	0.0115	3.3	Down	207181_s_at	CASP7	0.0479	2.08	Down
204554_at	PPP1R3D	0.0224	3.16	Down	204972_at	OAS2	0.0041	2.07	Down
219994_at	APBB1IP	0.0235	3.05	Down	203689_s_at	FMR1	0.0057	2.06	Down
225291_at	PNPT1	0.0068	3.05	Down	205202_at	PCMT1	0.0228	2.06	Down
216020_at	IFIH1	0.0015	2.95	Down	201786_s_at	ADAR	0.0403	2.04	Down
223434_at	GBP3	0.0194	2.86	Down	217886_at	EPS15	0.0119	2.04	Down
213361_at	TDRD7	0.0141	2.85	Down	207332_s_at	TFRC	0.0255	2.04	Down
214710_s_at	CCNB1	0.0039	2.71	Down	204822_at	TTK	0.0138	2.04	Down
236782_at	SAMD3	0.0260	2.69	Down	204820_s_at	BTN3A2	0.0439	2.02	Down
203925_at	GCLM	0.0408	2.67	Down	201921_at	GNG10	0.0327	2.02	Down
211597_s_at	HOPX	0.0083	2.67	Down	222608_s_at	ANLN	0.0168	2.01	Down
221287_at	RNaseL	0.0257	2.65	Down	208405_s_at	CD164	0.0384	2.01	Down
202687_s_at	TNFSF10	0.0443	2.58	Down	205077_s_at	PIGF	0.0310	2.01	Down
218085_at	CHMP5	0.0021	2.57	Down	206488_s_at	CD36	0.0859	2.01	Down

FC: Fold change, RG: Regulation.

#### PRRs signaling are the most impacted pathways in LBW newborns

**Figure 6:**
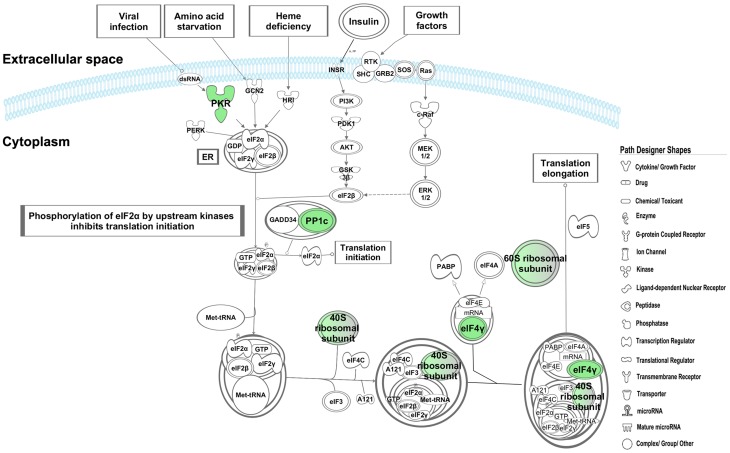
EIF2 signaling pathway. The down-regulated genes in LBW newborns are represented in green color. The intensity of node colour indicates the degree of down-regulation. The uncolored nodes are the genes inferred by IPA from its knowledge base.

The effect of down-regulated ISGs on the biological processes and pathways, as observed following the IPA analysis, was seen to overlap partially (differing in number of molecules involved in a canonical pathway/biological function) with the result of the IPA analysis carried out using all the 1065 down-regulated genes. Of the 199 signaling pathways resulting from the analysis, the three most significantly affected canonical pathways in LBW newborns were i)*‘activation of IRF by cytosolic pattern recognition receptors’,* ii)*‘role of pattern recognition receptors in recognition of bacteria and viruses’,* and iii)*‘eukaryotic initiation factor2 (eIF2) signaling’*. It is to be noted that the first two pathways were the top pathways associated with the down-regulated ISGs. The first pathway was related with anti-viral immune response and showed the down-regulation of genes encoding for key protein like retinoic acid inducible gene-1 (RIG-1), melanoma-differentiation associated gene-5(MDA-5), ISG-15, interferon stimulating gene-54 (ISG-54), STAT-2, IRF-7, tank-binding kinase (TBK), and inhibitor of kappa B kinase-α (IKK-α), (Figure 2). Genes encoding different PRRs such as transmembrane complement component 5a receptor (C5aR) and TLRs-1,-5, -8, as well as cytosolic RIG-1 and MDA-5 were down-regulated in the second pathway (Figure 3). In eIF2 signaling, protein kinase R (PKR)/eukaryotic translation initiation factor 2-alpha kinase 2 (EIF2AK2), protein phosphatase-1 catalytic subunit beta isozyme (PPIcB), eukaryotic translation initiation factor-4 gamma-2 (EIF4G2) were down-regulated along with ribosomal protein family genes (Figure 6).

#### Infectious and respiratory diseases are the most significantly associated bio-functions in LBW newborns

The functional analysis of 1065 down-regulated gene dataset identified biological functions that were most significantly associated with the down-regulated genes in LBW newborns (Table S3). *Respiratory infectious diseases* were significantly affected bio-function in LBW newborns. Severe acute respiratory syndrome (SARS) and bleeding of lungs were important diseases associated with LBW newborns. The important down-regulated genes associated with respiratory infectious diseases were leukotriene B4 receptor (LTB4R), cathelicidin antimicrobial peptide (CAMP), lactotransferrin (LTF), BPI, TLR-8, RIG-1 and MDA-5. Other infections such as flavivirus, hepatitis C virus, HIV and infection of mammalian were also the prominent infectious diseases found to be related with down-regulated genes in LBW newborns. The important down-regulated genes in LBW newborns associated with these infectious diseases were TLR-8, RIG-1 and MDA-5. *Inflammatory response* was an important biological function involving immune response, cell movement of neutrophils, antimicrobial response, and anti-viral response as important sub-categories. Other important bio-functions associated with the down-regulated genes in LBW newborns were *antigen presentation*, *hematological system development and function*, and *immune cell trafficking* which were primarily involved in activation, infiltration, chemotaxis, and recruitment of leukocytes. The important down-regulated genes associated with these functions were chemokines C-X3-C motif receptor 3 (CXCR3), C5aR, chemokines C-C motif receptor 1(CCR1), chemokine C-C motif ligand 4 (CCL4) and chemokines C-C motif receptor 2 (CXCR2).

#### Antimicrobial response is associated with the top networks generated by down-regulated genes in LBW newborns

A total of 25 networks with the highest scores and focus molecules were identified by IPA network analysis of 1065 down-regulated genes in LBW newborns. Network 1 was comprised of genes related to *antimicrobial response* (31 focus genes; IPA score of 43) (Figure S1). The main bio-functions associated with the down-regulated genes implicated in this network were innate immune response, antiviral response, inflammatory response of the cell, replication of RNA virus. Most of the genes in this network were ISGs and were common to those present in top canonical pathways and functions. IRF7 was hub gene around which most of the genes associated with antiviral responses interacted with each other. Network 4 also included genes involved in *antimicrobial response* (29 focus genes; IPA score of 38) (Figure S1). Down-regulated genes implicated in this network were found to be involved in activation of myeloid, leukocyte, cytotoxic T cells and lymphocytes cells. Granzymes (GZM-A, B, H, K) and BPI were the key genes involved in the antimicrobial response in this network.

### Up-regulated data set analysis

Analysis of up-regulated data set failed to reveal any significant association with immunological pathways and biofunctions and therefore was beyond the scope of this study. IPA analysis of up-regulated genes data set generated top pathways like *antigen presentation pathway, allograft rejection signaling, graft versus host disease signaling*. Major histocompatibility complex-α and -β, and tumor necrosis factor-α (TNF-α) were the only differentially expressed genes in LBW newborns which were present in these pathways. Top bio-functions affected due to up-regulation of genes in LBW newborns include *dermatological disease and conditions*, *immunological disease*, *inflammatory disease inflammatory response*, and *connective tissue disorders*. TNF-α and another gene high-mobility group protein B1 both of which are related with innate immune system, were found to be up-regulated in LBW newborns. This was an unexpected observation that needs further in-depth investigation. Since very few up-regulated genes related with immune functions are present in the top pathways and top bio-functions therefore, the significance of the association of these up-regulated genes in the data set with its pathways and bio-function gets subsided. Top most networks inferred by IPA were not immune response related and were thus not central to the interest of this study.

### Validation by real time RT-PCR

**Figure 7:**
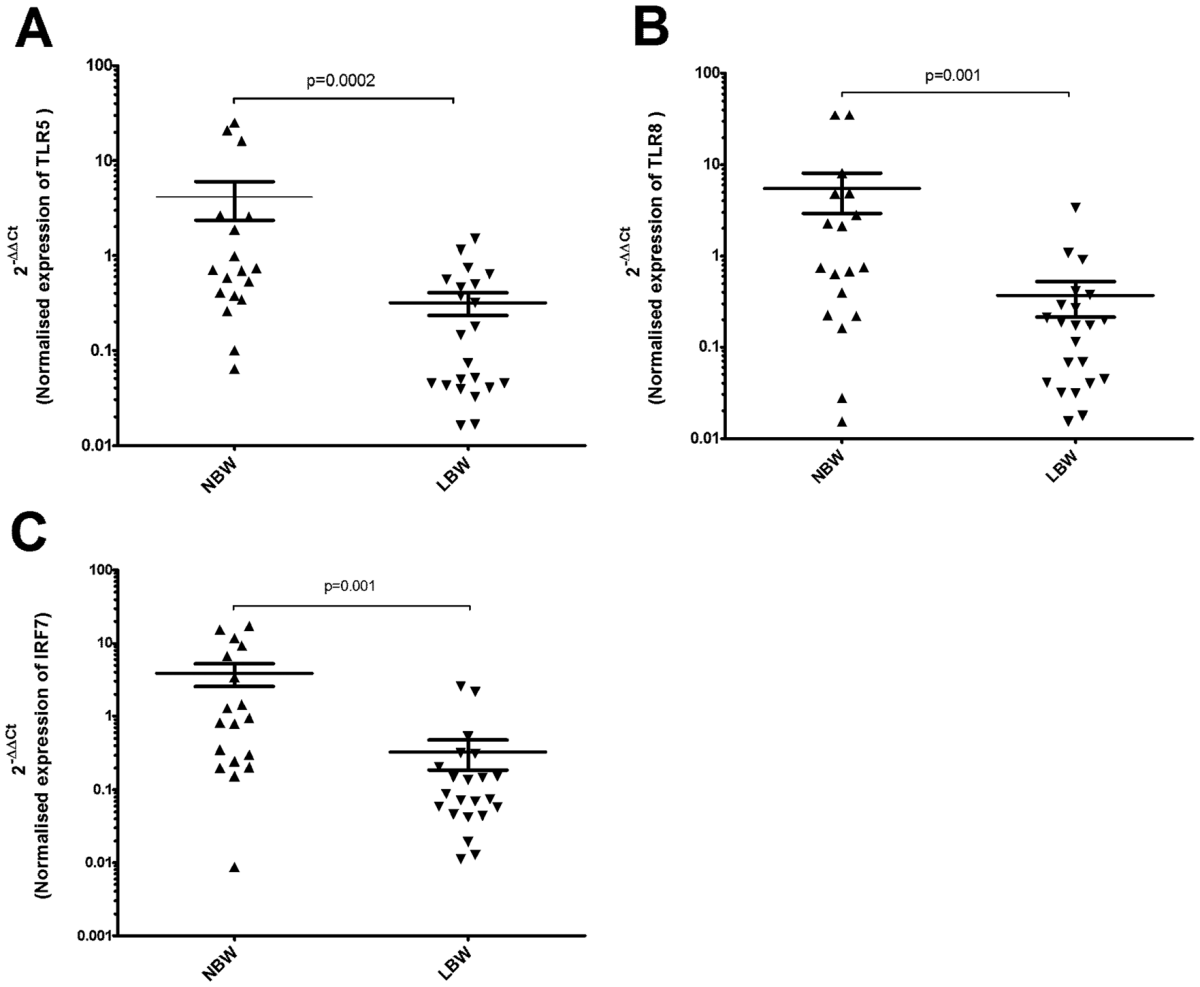
Quantitative Real-Time PCR validation of differentially expressed genes. Down regulation of (A) TLR-5 mRNA expression (**B**) TLR-8 mRNA expression (C) IRF-7 mRNA expression in LBW newborns compared to NBW newborns. The y-axis is a log_10_ scale. Black up pointing triangle (▴) denotes NBW samples (n = 22) and black down pointing triangle (▾) denotes LBW (n = 18) samples. The significant difference in mRNA level expression between LBW and NBW newborns is represented as p<0.05.

Three genes TLR-5, TLR-8, and IRF-7 that were under expressed in LBW newborns, were selected for validation of microarray data using quantitative real time RT-PCR analysis of cord blood RNA. The basis for selection of these genes was their involvement in the most of significantly affected pathways and function like *role of pattern recognition receptors in recognition of bacteria and viruses*, *activation of IRF by cytosolic pattern recognition receptors* and *infectious diseases*. All 8 LBW samples and one NBW samples (chosen on the basis of RNA availability) used in microarray assay were also subjected to real-time RT-PCR (Table S1). To compensate for the relatively small number of samples (LBW; 8 and NBW; 4) analyzed by microarray, we performed the real time RT-PCR validation on a larger group by including additional samples (LBW; 22, NBW; 18). The fold-changes in expression of these genes determined by real time RT-PCR were in concordance with microarray result with their expression levels significantly lower in the LBW newborns (Figure 7). Based on our real-time PCR data we found that gender of the newborn, age of mother, and type of delivery did not result into differences in transcription of the genes validated (data not shown).

### TLR-5 and TLR-8 protein validation by western blot analysis

**Figure 8:**
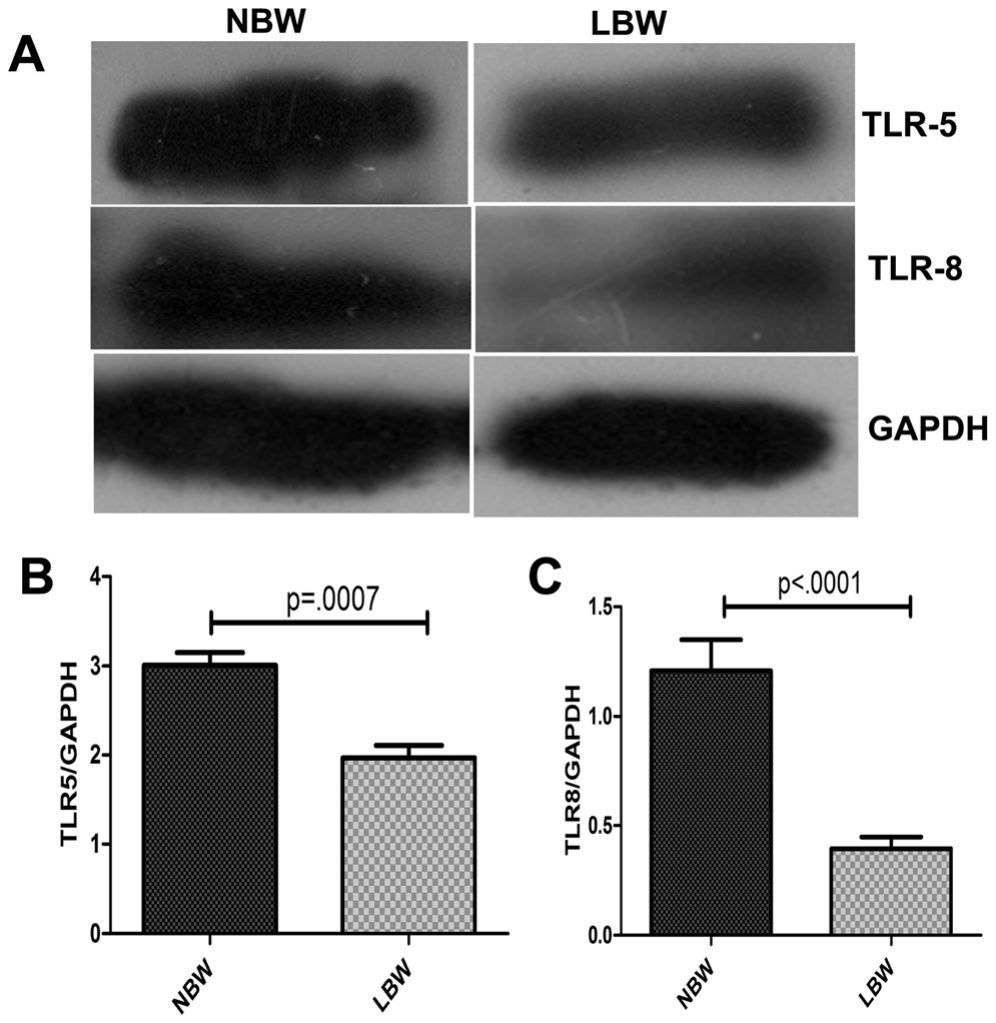
Representative western blot figure for expression of TLR5 and TLR8 levels. (A) Expression of TLR5 and TLR8 in NBW and LBW newborns. Densitometric analysis of (B) TLR5 and (C) TLR8 gene normalised against GAPDH in LBW and NBW newborns. The difference in TLR5 and TLR8 expression levels between LBW and NBW newborns was significant; p<0.05, (LBW = 12, NBW = 12).

TLR-5 and TLR-8 genes which were found to be down-regulated in LBW newborns by microarray and real-time RT-PCR were subjected to protein level validation by western blotting. Densitometric analysis of the western blot results revealed that the expression of TLR-5 and TLR-8 proteins were also significantly down-regulated in LBW newborns (Figure 8).

### Production of IFN-α is low in response to TLR-8 signaling in LBW newborns

**Figure 9:**
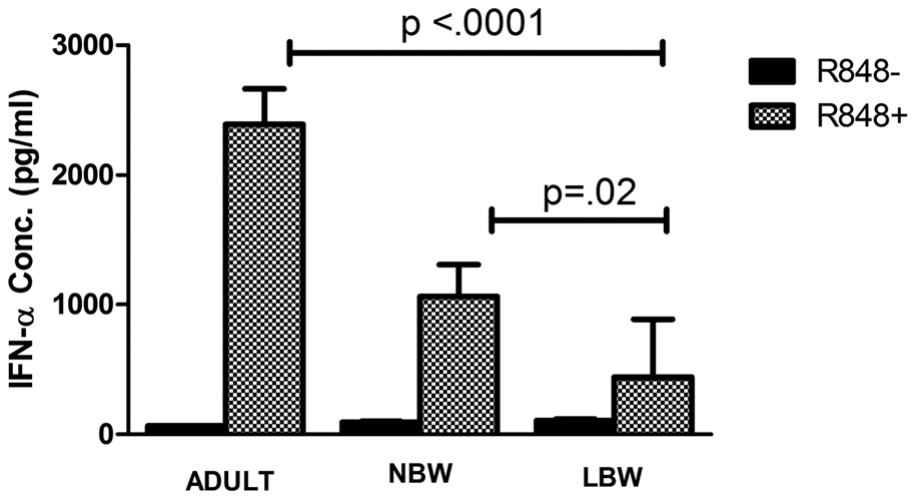
Quantitative measurement of human IFN-α by ELISA. Cord blood from 18 LBW and 18NBW newborns and peripheral blood from 8 adults were cultured with R-848 for 18 hrs and IFN-α released in culture supernatant was measured. There was significant difference in the production of IFN- α between LBW vs NBW newborns and adults (p<0.05).

Cord blood of NBW and LBW newborns and peripheral blood of adults were stimulated by R-848 for IFN-α production. IFN-α production in response to R-848 was significantly low in LBW newborns (Mean IFN-α 437 pg/ml; range 153–1727 pg/ml) compared to both NBW newborns (Mean IFN-α 1060 pg/ml; range 179–3792 pg/ml; p<0.05) and adults (Mean IFN-α 2389 pg/ml; range 1208–3228 pg/ml; p<0.001) (Figure 9).

### Release of BPI protein is low in response to TLR-8 signaling in LBW newborns

**Figure 10:**
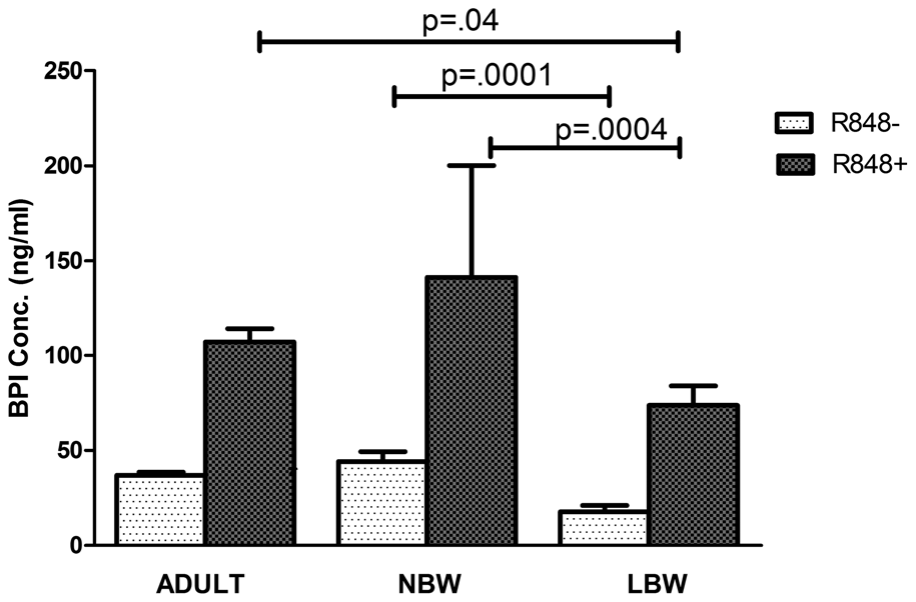
Quantitative measurement of human BPI by ELISA. Cord blood from 18 LBW and 18 NBW newborns and peripheral blood from 8 adults were cultured with R-848 for 18 hrs and BPI protein released in culture supernatant were measured. There was significant difference in the BPI level between LBW and NBW newborns (p<0.05) and between LBW newborns and adults (p<0.05) both under induced and uninduced conditions.

We report significantly lesser release of BPI protein in response to R-848 from LBW newborns (Mean BPI 73.89 ng/ml; range 21.9–167.4 ng/ml) compared to NBW newborns (mean BPI 141.22 ng/ml; range 59.37–232.8 ng/ml; p = 0.0004.) (Figure 10). We also noticed that BPI protein level was significantly lower in the uninduced cord blood culture supernatant of LBW newborns (mean BPI 17.5 ng/ml; range 2.5–59.7 ng/ml) compared to that of NBW newborns (Mean BPI 44.1 ng/ml, range 16.2–84.5 ng/ml; p = 0.0001) (Figure 10). Intriguingly, BPI protein release was significantly higher in adults (Mean BPI 107.09 ng/ml; range 78.1–120.6 ng/ml; p = 0.04) compared to the LBW newborns but was lower than the BPI protein levels released from cord blood of NBW newborns.

## Discussion

In this study, we analyzed global gene expression of cord blood leukocytes of LBW newborns, and identified 1391differentailly expressed genes in LBW newborns. This is the first study that we are aware of comparing the whole blood transcriptome of LBW newborns with those of NBW newborns and identified the genes that might be responsible for impaired innate immune responses in the LBW newborns. The distinct innate immune system of the LBW newborn is possibly the outcome of weak signaling of genes which work in an integrated manner. Therefore, any defect in their interaction may lead to an impaired innate immune response, thus, predisposing these newborns to various viral and bacterial infections.

Down-regulation of a large spectrum of PRRs involved in the *‘role of pattern recognition receptors in recognition of bacteria and viruses’* pathway suggest impaired innate immune signaling against the invading pathogens in the LBW newborns. C5aR (cytosolic PRR) is the receptor for an anaphylatoxin C5a which chemoattracts granulocytes particularly the neutrophils at the site of infection and contributes to the pathogenesis of inflammation and sepsis [21], [22]. Reduced expression of C5aR on neonatal neutrophils contributes to poor chemotaxis and transmigration compared to adult neutrophils [23]. TLR-1 and -5 (extracellular PRRs) mainly recognize bacterial products, whereas TLR-8 (intracellular PRR) can detect both bacterial and viral products [24], [25]. Although basal TLR expression of full-term newborns blood monocytes is comparable to that of adults [9], [26] the status of expression of TLRs among term LBW newborns has not been studied so far. In this study, we found that the basal expression of TLR-1, TLR-5, and TLR-8 at mRNA and TLR-5 and TLR-8 at protein level is down-regulated in LBW newborns as compared to NBW newborns. Although direct evidence of TLR-1and TLR-5-mediated protection of newborns against infection is not available, a study in adults reports that individuals with low expression of TLR-1 were hypo-responsive to vaccination against Lyme disease [27] and TLR-5 signaling has a protective role against pneumococcal infection in mice [28]. TLR-8 agonists are potent inducer of cytokines in newborns and implicated as a candidate vaccine adjuvant in both early life and adulthood [29], [30]. Decreased TLR-8 expression in monocytes of respiratory syncytial virus (RSV)-infected newborns dampens early anti-viral cytokine production [31]. Low production of BPI protein as observed in LBW newborns might be due to the impaired TLR-8 signaling as TLR-8 is known to induce the type1 IFN such as IFN-β through IRF7 [25] which in turn induces BPI protein in mice [32]. Thus, our observation that TLR8, IFNs, and BPI are down-regulated in the LBW newborns is supported by the above studies. Defective cytokine responses due to impaired TLR signaling correlates with heightened risk from excessive inflammation and clinical patterns of susceptibility to disease in prematurely born infants [33]. Furthermore, diminished expression of TLRs and their signaling increases severity of diseases [31], [34] and seems to be an important immune evasion mechanism for some microbial pathogens [35].

*‘Activation of IRF by cytosolic pattern recognition receptors’* pathway has functional consequences of maintaining the anti-viral immune response. IRFs are key transcription factors involved in PRRs and IFN signaling and their reduced expression could have direct influence on the release of pro-inflammatory cytokines. RIG-1 and MDA-5 are cytosolic PRRs that sense viral infections through recognition of viral dsRNA leading to activation of TBK and IKK-α and result in induction of type I IFNs through activation of IRF-7 transcription factor. Induction of type I IFNs, in turn, induces the apoptosis of virus-infected cells and provides cellular resistance to virus infection [36], [37]. Additionally, activation of type I IFNs leads to transcription of ISGs like ISG-15 and ISG-54 through JAK/STAT pathway that result in an antiviral response in infected and neighboring cells [38], [39], [40]. The reduced production of IFN-α significantly increases the risk of recurrent wheezing in newborn [41]. The impaired IFN-α production is reported in cord blood and in the blood of 4-day's old baby at mRNA and protein levels [42]. Furthermore, defective expression of type I IFN genes are associated with impaired translocation of IRF7 in cord blood [43]. Down-regulation of PRRs and IRFs implicated in this pathway could impair cytosolic recognition of viral particles and may lead to low production of IFNs as we have observed in LBW newborns the low production of IFN-α in response to R-848 in LBW newborns. This suggest that the down-regulation of these PRRs and their effector molecules have potential role in the outcome of infections in LBW newborns.

The *‘eIF2 signaling’*, related with the *‘activation of IRF by cytosolic pattern recognition receptors’* pathway, is induced upon exposure of the cell to a variety of stressors including viruses. It serves to inhibit ribosomal translation of cellular and viral proteins, ultimately terminating viral replication in the cell and inducing apoptosis [44]. PKR, a member of eIF2 kinase family, is activated by dsRNA, and arrests cellular and viral protein synthesis by limiting viral replication through phosphorylation of the α subunit of eIF-2 [45], [46]. Given that eIF-2α phosphorylation is crucial for virus-induced translation termination [47] and apoptosis [48], [49] down-regulation of PKR and other members of eIF2 signaling may provide a favorable ground for progression of viral infection in the LBW newborns. Additionally, the reduced expression of a large number of small and large ribosomal proteins implicated in eIF2 signaling may suggest severe translational dysfunction in the host cell resulting in lower turnover of cellular proteins which may be an indication of developmental insufficiency.

We found an extensive down-regulation of ISGs and *IFN signaling* in the LBW newborns which has not been previously reported. IFNs are multifunctional cytokines that mediate their effects via transcription of ISGs and play an essential role in host immunity by inhibiting the replication and spread of viral, bacterial, and parasitic pathogens [50]. The down-regulated ISGs in LBW newborns such as PKR/EIF2AK2, myxovirus resistance-1 (MX-1), ribonuclease L (RNaseL), 2′-5′-oligoadenylate synthetase (OAS)-1, -2, and 2′-5′-oligoadenylate synthetase-like (OASL) are some of the best studied ISGs and function as antiviral effectors [51]. IFIT-1, IFIT-2, IFIT-3, and IFIT-5, which were found to be the most down-regulated genes in LBW newborns, are induced in response to type I and type II IFNs against viral infections [52], [53]. It has been demonstrated that IFIT-1 recognizes 5′-triphosphate RNA of viral origin and is involved in efficient antiviral activity in the presence of all three family members (IFIT-1, IFIT-2 and IFIT-3) forming an IFN dependent multi-protein complex [53]. Furthermore, IFIT-1 and IFIT-2 have been reported to regulate virus-triggered type I IFN signaling [54] and LPS induced excessive TNF-α expression [55] respectively, thus, offering protection to host against cellular damage caused by excessive release of cytokines. Interestingly, IFIT's ability to recognize the nucleic acid from a variety of yet-unidentified microbes has been proposed as analogous to the role of TLRs [53]. The antiviral role of IFITs and other ISGs has not been studied earlier in newborns. The observed down-regulation of these genes in the LBW newborns might help explain the severe outcome of viral infections compared to the NBW newborns. These findings may be extended to investigate the roles and interplay of ISGs in the weakened immune response in the LBW newborns.

Our finding of *infectious and respiratory diseases* associated with down-regulated genes in LBW newborns is in agreement with other studies [3], [7], [56]-[58]. Moreover, the risk of infections in LBW newborns is not only very high in the perinatal period but is also linked to onset of long term chronic diseases in the adulthood like respiratory complications including wheezing, coughing and pulmonary infections [58]–[61]. LTB4R, CAMP, LTF, and BPI genes were found to be implicated in SARS affected patients [62]. Nucleic acid-sensing PRRs including RIG-1, MDA-5, TLR-8 were found to be associated with acute bronchiolitis in infants [63]. Decreased TLR-8 expression has been correlated with increased lower respiratory tract RSV-infection in the infants [31]. Down-regulated genes in the *infectious disease* category are involved with replication of Flavivirus, Reovirus, Picorna virus, Orthomyxovirus etc. [64], [65]. The PRRs play important roles in managing these viral infections as RIG-1 and MDA-5 are reported to recognize viral components of Japanese encephalitis virus, dengue virus, West Nile virus, which belong to the Flaviviridae family, and induce type I IFNs [66]–[68].

This study found that *inflammatory response, antigen presentation, hematological system development,* and *immune cell trafficking* were important biological processes affected in LBW newborns. The possible mechanism involved in these processes include activation, infiltration, chemotaxis, and recruitment of various leukocytes like myeloid, granulocytes, neutrophils, and macrophages at the sites of infections. The observed down-regulation of chemokine (CCL4) and chemokine receptors (CCR1, CXCR2, CX3CR1, C5aR) could impair the proper inflammatory response at the site of infection as they play a crucial role in immune cell trafficking [69]. C5aR, CCR1 and CXCR2 regulate leukocyte trafficking [22], [70], [71]. Similarly, CCL4 which acts on neutrophils, monocytes, and lymphocytes, plays a pivotal role in the development, function, and homeostasis of the immune system of the host [70]. Furthermore, CXCR2 is predominantly responsible for neutrophil recruitment to the site of infection [71] whereas, the CX3CR1 receptor regulates infiltration of peripheral macrophages and, plays important role in immune surveillance [72]. This suggests that the down-regulation of chemokines and chemokine receptors may cause the impaired recruitment of leukocytes at the site of infection.

Reduced expressions of granzymes and BPI in LBW newborns are also linked with antimicrobial responses. Granzymes are produced by cytotoxic T cells and natural killer cells and, are released upon interaction with target cells. To date, five different granzymes have been described in humans: granzymes A, B, H, K and M [73]. Wang et al noted low expression of granzyme B due to reduced activity of cord blood NK cells [74]. This finding together with our result provides support for suggesting that the LBW newborns may have low NK cell activity reflected by decreased expression of granzymes and granzyme B in particular. BPI, an important antimicrobial polypeptide, is derived from primary granules of neutrophils [75], and to a lesser extent from eosinophils [76]. BPI has high affinity for the endotoxin of Gram-negative bacteria and exhibits a cytotoxic activity against them [77]–[79]. BPI protein helps the host body in fighting with gram negative bacteria by targeting its opsonization, by neutralizing LPS mediated inflammatory responses, and through microbicidal activity [80]. Neonatal cord blood neutrophils have less (3–4 fold) intracellular BPI than the adults and this deficiency is correlated with diminished neutrophil activity against *E.coli* K1/r [81]. We have shown that production of BPI protein was significantly lower in response to R-848 in LBW newborns compared to NBW newborns. Moreover, BPI protein in unstimulated culture supernatant was also significantly lower in LBW newborns compared to NBW newborns however, the unstimulated culture supernatant of NBW newborns and adults were comparable. This may be an indication of impaired immune status of LBW newborns. The down-regulation of BPI detected in LBW newborns is suggestive of weakened neutrophil functions in LBW newborns.

In conclusion, this microarray study has identified some unique molecular pathways and genes that may have a predominant role in determining the strength of the immune responses in the LBW newborns against microbial infections. Ongoing functional investigations focused on the deficient immune factors identified through this study may provide valuable information on the possible underlying mechanisms of increased susceptibility to infection in the term LBW newborns. Although dangers of administering new molecules to newborns are of utmost importance, development of innate immune system mediated adjunctive therapy targeting the genes identified in these pathways and biofunctions may help protect the LBW newborns from the enhanced susceptibility and severity of infections. Further, research into PRRs especially TLR-mediated immunity in this area may have great relevance to clinical practice.

## Supporting Information

Figure S1 **Top four networks of down-regulated genes in LBW newborns.** In the screen shot view generated by IPA, columns show: All molecules in each network, IPA score, total number of focus genes found, and top functions. The downward arrow (↓) in green indicates down-regulated focus genes associated with LBW newborns in a network.
(TIF)

Table S1 **Demographic characteristics of study population.**
(DOCX)

Table S2 **List of all differentially expressed genes in LBW Newborns.**
(XLSX)

Table S3 **Top bio-functions associated with down-regulated genes in LBW newborns.**
(DOCX)
