# Supplementary figures and images for: Decreased Pattern Recognition Receptor Signaling, Interferon-Signature, and Bactericidal/Permeability-Increasing Protein Gene Expression in Cord Blood of Term Low Birth Weight Human Newborns
Source: PLoS One. 2013 Apr 23;8(4):e62845. doi: 10.1371/journal.pone.0062845 (PMC3633842; doi:10.1371/journal.pone.0062845)

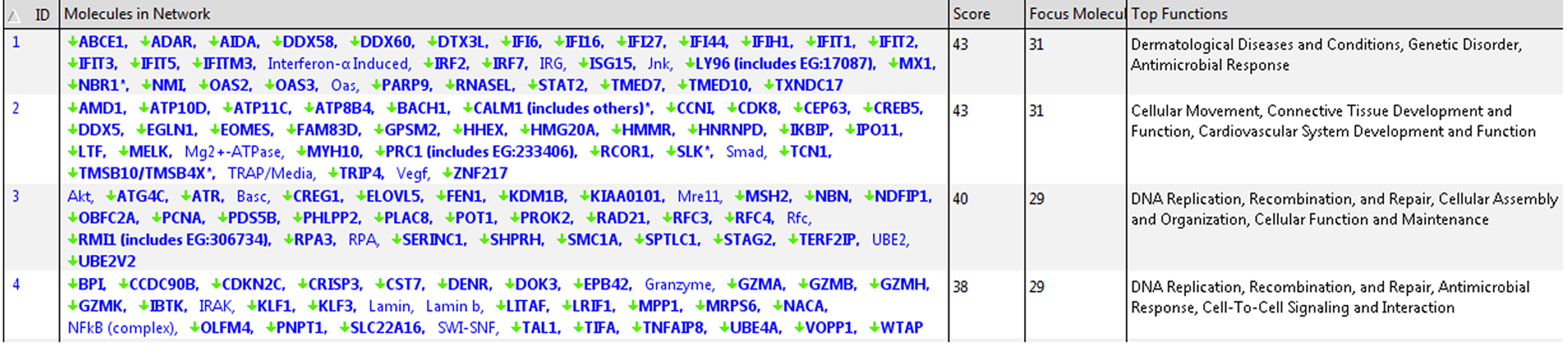

Supplement: Figure S1 — Top four networks of down-regulated genes in LBW newborns. In the screen shot view generated by IPA, columns show: All molecules in each network, IPA score, total number of focus genes found, and top functions. The downward arrow (↓) in green indicates down-regulated focus genes associated with LBW newborns in a network. (TIF) [file pone.0062845.s001.tif]
