# Supplementary material for: Decreased Pattern Recognition Receptor Signaling, Interferon-Signature, and Bactericidal/Permeability-Increasing Protein Gene Expression in Cord Blood of Term Low Birth Weight Human Newborns
Source: PLoS One. 2013 Apr 23;8(4):e62845. doi: 10.1371/journal.pone.0062845 (PMC3633842; doi:10.1371/journal.pone.0062845)
Supplement: Table S1 — Demographic characteristics of study population. (DOCX) [file pone.0062845.s002.docx]

Table S1. Demographic Characteristics of Study Population.

| S.No. | Group | Sample ID | Gender | Gestation Age (Weeks + Days) | Type of  delivery | Birth  weight (g) | Assay |
| --- | --- | --- | --- | --- | --- | --- | --- |
| 1 | LBW | E1 | M | 38+4 | EMCS | 2190 | MA/RT-PCR |
| 2 | LBW | E2 | F | 39+2 | SVD | 2400 | MA/RT-PCR |
| 3 | LBW | E3 | M | 37 | EMCS | 2380 | MA/RT-PCR |
| 4 | LBW | E4 | M | 37+3 | SVD | 2020 | MA/RT-PCR |
| 5 | LBW | E5 | M | 37 | EMSC | 1825 | MA/RT-PCR |
| 6 | LBW | E6 | M | 37 | EMCS | 1725 | MA/RT-PCR |
| 7 | LBW | E7 | M | 37 | EMCS | 1845 | MA/RT-PCR |
| 8 | LBW | E8 | F | 37+6 | EMCS | 1860 | MA/RT-PCR |
| 9 | LBW | E9 | M | 37 | SVD | 2040 | RT-PCR |
| 10 | LBW | E10 | F | 42+1 | EMCS | 2100 | RT-PCR |
| 11 | LBW | E11 | M | 38+6 | EMCS | 2160 | RT-PCR, WB |
| 12 | LBW | E12 | F | 37+5 | EMCS | 2280 | RT-PCR, WB |
| 13 | LBW | E13 | F | 39+6 | SVD | 2480 | RT-PCR, WB |
| 14 | LBW | E14 | F | 38+2 | EMCS | 2240 | RT-PCR, WB |
| 15 | LBW | E15 | M | 39+5 | EMCS | 2300 | RT-PCR, WB |
| 16 | LBW | E16 | F | 37 | SVD | 1900 | RT-PCR, WB |
| 17 | LBW | E17 | M | 37+4 | SVD | 1950 | RT-PCR, WB |
| 18 | LBW | E18 | F | 38 | SVD | 2020 | RT-PCR, WB |
| 19 | LBW | E19 | F | 37 | SVD | 1825 | RT-PCR, WB |
| 20 | LBW | E20 | F | 40+4 | EMCS | 2240 | RT-PCR, WB |
| 21 | LBW | E21 | F | 35+6 | SVD | 2120 | RT-PCR, WB |
| 22 | LBW | E22 | F | 39+1 | SVD | 2300 | RT-PCR, WB |
| 23 | LBW | E23 | M | 38+1 | EMCS | 2200 | IFN- α and BPI ELISA |
| 24 | LBW | E24 | M | 37 | EMCS | 2150 | IFN- α and BPI ELISA |
| 25 | LBW | E25 | M | 37+2 | SVD | 2300 | IFN- α and BPI ELISA |
| 26 | LBW | E26 | F | 37+6 | SVD | 2100 | IFN- α and BPI ELISA |
| 27 | LBW | E27 | M | 38+2 | SVD | 2350 | IFN- α and BPI ELISA |
| 28 | LBW | E28 | F | 37 | EMCS | 2100 | IFN- α and BPI ELISA |
| 29 | LBW | E29 | F | 38 | SVD | 2200 | IFN- α and BPI ELISA |
| 30 | LBW | E30 | F | 38+3 | SVD | 2300 | IFN- α and BPI ELISA |
| 31 | LBW | E31 | M | 37+1 | EMCS | 2100 | IFN- α and BPI ELISA |
| 32 | LBW | E32 | F | 37+6 | EMCS | 2200 | IFN- α and BPI ELISA |
| 33 | LBW | E33 | M | 38+5 | EMCS | 2250 | IFN- α and BPI ELISA |
| 34 | LBW | E34 | M | 38+4 | EMCS | 2300 | IFN- α and BPI ELISA |
| 35 | LBW | E35 | F | 38+3 | SVD | 2350 | IFN- α and BPI ELISA |
| 36 | LBW | E36 | F | 37+5 | SVD | 2250 | IFN- α and BPI ELISA |
| 37 | LBW | E37 | F | 39+4 | EMCS | 2100 | IFN- α and BPI ELISA |
| 38 | LBW | E38 | M | 38+2 | EMCS | 2150 | IFN- α and BPI ELISA |
| 39 | LBW | E39 | F | 37+5 | EMCS | 2200 | IFN- α and BPI ELISA |
| 40 | LBW | E40 | M | 37 | SVD | 2300 | IFN- α and BPI ELISA |
| 41 | NBW | C1 | F | 38+5 | SVD | 3020 | MA |
| 42 | NBW | C2 | M | 37 | EMCS | 3000 | MA |
| 43 | NBW | C3 | F | 39+5 | SVD | 2900 | MA |
| 44 | NBW | C4 | M | 39+4 | SVD | 3500 | MA/RT-PCR |
| 45 | NBW | C5 | F | 37 | EMCS | 3000 | RT-PCR |
| 46 | NBW | C6 | M | 38+3 | EMCS | 2720 | RT-PCR |
| 47 | NBW | C7 | M | 38+4 | EMCS | 3020 | RT-PCR |
| 48 | NBW | C8 | F | 39+4 | SVD | 2900 | RT-PCR |
| 49 | NBW | C9 | F | 39+3 | EMCS | 2940 | RT-PCR |
| 50 | NBW | C10 | F | 37 | SVD | 2600 | RT-PCR, WB |
| 51 | NBW | C11 | F | 39+4 | EMCS | 2760 | RT-PCR, WB |
| 52 | NBW | C12 | F | 39+1 | SVD | 2540 | RT-PCR, WB |
| 53 | NBW | C13 | M | 40+1 | EMCS | 2600 | RT-PCR, WB |
| 54 | NBW | C14 | F | 41 | SVD | 2620 | RT-PCR, WB |
| 55 | NBW | C15 | F | 39+6 | SVD | 2880 | RT-PCR, WB |
| 56 | NBW | C16 | M | 38+4 | SVD | 2750 | RT-PCR, WB |
| 57 | NBW | C17 | M | 39+0 | EMCS | 2700 | RT-PCR, WB |
| 58 | NBW | C18 | M | 40+1 | EMCS | 3500 | RT-PCR, WB |
| 59 | NBW | C19 | M | 39 | SVD | 3550 | RT-PCR, WB |
| 60 | NBW | C20 | F | 38+5 | SVD | 3250 | RT-PCR, WB |
| 61 | NBW | C21 | F | 40 | SVD | 3000 | RT-PCR, WB |
| 62 | NBW | C22 | M | 38+5 | EMCS | 2950 | IFN-α and BPI ELISΑ |
| 63 | NBW | C23 | M | 39 | EMCS | 3000 | IFN-α and BPI ELISΑ |
| 64 | NBW | C24 | M | 38+3 | EMCS | 2800 | IFN- α and BPI ELISA |
| 65 | NBW | C25 | F | 38+6 | SVD | 3200 | IFN- αand BPI ELISA |
| 66 | NBW | C26 | M | 39+2 | EMCS | 2800 | IFN- α and BPI ELISA |
| 67 | NBW | C27 | F | 37+5 | SVD | 3000 | IFN- α and BPI ELISA |
| 68 | NBW | C28 | F | 38 | SVD | 2800 | IFN- α and BPI ELISA |
| 69 | NBW | C29 | F | 38+2 | SVD | 3000 | IFN- α and BPI ELISA |
| 70 | NBW | C30 | M | 38+3 | EMCS | 2900 | IFN- α and BPI ELISA |
| 71 | NBW | C31 | F | 38+5 | SVD | 2700 | IFN- α and BPI ELISA |
| 72 | NBW | C32 | F | 39 | EMCS | 2900 | IFN- α and BPI ELISA |
| 73 | NBW | C33 | M | 39 | SVD | 2800 | IFN- α and BPI ELISA |
| 74 | NBW | C34 | F | 39+3 | SVD | 3300 | IFN- α and BPI ELISA |
| 75 | NBW | C35 | F | 38+5 | SVD | 3500 | IFN- α and BPI ELISA |
| 76 | NBW | C36 | M | 37+6 | SVD | 2700 | IFN- α and BPI ELISA |
| 77 | NBW | C37 | F | 38+4 | EMCS | 2800 | IFN- α and BPI ELISA |
| 78 | NBW | C38 | M | 39+1 | SVD | 2850 | IFN- α and BPI ELISA |
| 79 | NBW | C39 | F | 38+2 | EMCS | 2900 | IFN- α and BPI ELISA |
| 80 | Adult | A1 | M | N/A | N/A | N/A | IFN- α and BPI ELISA |
| 81 | Adult | A2 | F | N/A | N/A | N/A | IFN- α and BPI ELISA |
| 82 | Adult | A3 | F | N/A | N/A | N/A | IFN- α and BPI ELISA |
| 83 | Adult | A4 | M | N/A | N/A | N/A | IFN- α and BPI ELISA |
| 84 | Adult | A5 | F | N/A | N/A | N/A | IFN- α and BPI ELISA |
| 85 | Adult | A6 | F | N/A | N/A | N/A | IFN- α and BPI ELISA |
| 86 | Adult | A7 | M | N/A | N/A | N/A | IFN- α and BPI ELISA |
| 87 | Adult | A8 | F | N/A | N/A | N/A | IFN- α and BPI ELISA |

M: Male, F: Female; EMCS: Emergency Caesarian Section, SVD: Spontaneous Vaginal Delivery; MA: Microarray Analysis, RT-PCR: real time Reverse Transcriptase-PCR, WB: Western-blot.
