# Supplementary material for: Decreased Pattern Recognition Receptor Signaling, Interferon-Signature, and Bactericidal/Permeability-Increasing Protein Gene Expression in Cord Blood of Term Low Birth Weight Human Newborns
Source: PLoS One. 2013 Apr 23;8(4):e62845. doi: 10.1371/journal.pone.0062845 (PMC3633842; doi:10.1371/journal.pone.0062845)
Supplement: Table S3 — Top bio-functions associated with down-regulated genes in LBW newborns. (DOCX) [file pone.0062845.s004.docx]

Table S 3. Top bio-functions associated with down-regulated genes in LBW newborns.

| **Bio-Functions** | **p-value** | **Number of Molecules** |
| --- | --- | --- |
| *Infectious Disease* (Infection by virus, replication of virus, replication of RNA virus, replication of Influenza virus, infection of cells, replication of Influenza A virus, replication of Flaviviridae, replication of Hepatitis C virus, infection of mammalian, infection by bacteria). | 1.67E-17 - 6.04E-03 | 178 |
| *Respiratory Disease* (Severe acute respiratory syndrome, lung adenocarcinoma, lung tumor, bleeding of lung). | 1.67E-17 - 6.36E-03 | 69 |
| *Inflammatory Response* (Immune response, cell movement of neutrophils, antimicrobial response, antiviral response) | 1.24E-16 - 8.06E-03 | 145 |
| *Antigen Presentation* (Chemotaxis of neutrophils, chemotaxis of phagocytes, activation of phagocytes, immune response of macrophages, activation of neutrophils, immune response of phagocytes, chemo-attraction of neutrophils, chemo-attraction of phagocytes, binding of monocyte-derived macrophages) | 3.13E-06 - 4.38E-03 | 53 |
| *Hematological System Development and Function* (Cell, movement of granulocytes, proliferation of immune cells, chemotaxis of neutrophils, proliferation of lymphocytes, hematopoiesis, chemotaxis of leukocytes) | 1.14E-07 - 8.21E-03 | 140 |
| *Immune Cell Trafficking* (cell movement of neutrophils, cell movement of granulocytes, chemotaxis of neutrophils, chemotaxis of leukocytes, activation of leukocytes, infiltration of granulocytes, infiltration of myeloid cells, recruitment of myeloid cells, recruitment of granulocytes, recruitment of neutrophils) | 1.14E-07 - 8.13E-03 | 81 |
